# Who pays for clean air, and why?: Analyzing the impact of resource, planned behavior, and government/policy factors on the multidimensional willingness to pay for reducing the particulate matter

**DOI:** 10.1016/j.heliyon.2024.e34643

**Published:** 2024-07-14

**Authors:** Sehyeok Jeon, Seoyong Kim, Sohee Kim

**Affiliations:** a Social Science Research Institute, Ajou University, Suwon, 16499, Republic of Korea; b Department of Public Administration, Ajou University, Suwon, 16499, Republic of Korea

**Keywords:** PM (particulate matter), Multidimensionality of willingness to pay, Theory of planned behavior, Resource theory, Government/policy factor

## Abstract

This study aimed to explore the determinants of willingness to pay for fine particulate matter reduction. Previous studies were mostly based on simple causal models, with few or similar predictors affecting a single dimension of willingness to pay. This study adopted a multidimensional model, dividing willingness to pay into three categories: cost burden, benefit to the community and benefit to specific groups. The independent variables were resources, planned behavior, and government/policy factors, with a total of 12 variables. The analysis showed that, first, the determinant structure varied across the dimensions of willingness to pay. Second, facility resources, information, personal norms, social norms, perceived control, trust in government, policy satisfaction, policy preference, and policy knowledge had significant positive effects on willingness to pay. Third, policy satisfaction and social norms had high explanatory power for willingness to pay for cost burden; policy preference and personal norms for benefit to community; and policy knowledge, policy preference, and perceived control for benefit to specific groups.

## Highlights

• This study explores how the seventeen variables have impact on the willingness to pay for reducing the particulate matter.
• The analysis revealed that the determinant structure varied, depending on the dimension of the willingness to pay.
• Policy satisfaction and norms exhibited high power in explaining the willingness to pay for cost burden.
• Policy knowledge and norms had high power in explaining the pay for benefits to the community and specific groups.

## 1 Introduction

This study aimed to analyze the factors that influence the willingness to pay (WTP) for particulate matter (hereafter, PM)-related services. This study focused on the multidimensionality of causes and effects in a causal relationship. The rationale for this study is as follows:

First, a proactive approach to PM is required because it becomes the main target for public and government to cope and solve itself. PM generally refers to dust that is small enough to be invisible, and comprises sulfate, nitrate, carbon, metal compounds, and earth dust (minerals, etc.). The International Agency for Research on Cancer (IARC) under the World Health Organization designated fine dust as a Group 1 carcinogen. Additionally, a recent study confirmed very high levels of heavy metal content in PM, prolonged exposure to which can cause respiratory diseases (such as bronchitis and asthma), cardiovascular and skin diseases.

Recognizing of the dangers of fine dust, the Korean government has announced subsidies for scrapping diesel vehicles with emission level 5 and implemented preliminary fine dust reduction measures in metropolitan areas to tackle high concentration of fine dust in spring. Emergency reduction measures refer to short-term measures to improve air quality when high concentrations of fine dust persist for a certain period; the Special Act on Fine Dust Reduction and Management was enforced in February 2019. Emergency reduction measures are transmitted through emergency text messages, media, broadcasting, local government websites, and mobile apps (Air Korea) one day before the effective date, and the public should actively heed these measures. Central and local governments should strengthen crackdowns and inspections of emissions and illegal incineration to reduce air pollution and encourage public participation through publicity. Pollutant-emitting worksites should adjust their hours and rates of operation and implement reduction measures to improve the efficiency of pollution prevention facilities. The general public should use public transportation or practice national behavioral guidelines such as reducing energy use and implementing vehicle operation restrictions according to local ordinances, where emergency reduction measures are issued.

A new approach is being attempted to reduce the damage from PM, and the COVID-19 pandemic has made people more concerned than ever before about health and socioeconomic costs and benefits. Therefore, robust PM reduction and damage response policies are needed to improve people's quality of life and social welfare.

Second, there are additional costs associated with implementing particulate matter reduction policies, whether by government or external organizations, which can be difficult to accurately estimate. In response, the public will make judgments about how much they can afford to pay in relation to whether they support the policy. Understanding willingness to pay, which is the basis for these judgments, is essential for the successful implementation of policies. Therefore, estimating and investigating willingness to pay is a proactive step in the context of fine particulate matter reduction.

Third, while external groups and organizations should respond actively to PM issues, individual participation is crucial. Everyday transportation, consumption, and leisure activities are among the main sources of PM. Therefore*, due to the nature of the PM reduction policy, the participation and intention of the general public is very important.* However, few studies have analyzed the factors that influence an individual's response to PM. Research on the extent to which the expected response involves paying for the problem to be solved. Therefore, this study examines how different causal factors affect the willingness to pay for PM reduction. This study will analyze how the three independent factors (i.e., resources, planned behavior, and government/policy) affect the multidimensionality of the willingness to pay for reducing PM. This study constructs the willingness to pay by constructing three dimensions, instead of just one.

In the theoretical background that follows, this study reviews the literature of willingness to pay and present hypotheses. The next section describes the research design and methods to measure each factor. In section 4, the hypotheses are analyzed by performing descriptive pattern, correlation, and regression analyses. Section 5 discusses the implications of this study based on the results as well as future research directions, while Section 6 concludes the study.

## 2 Theory

### 2.1 Multidimensionality of willingness to pay

Willingness to pay is a measure of the value that an individual accords to consumption or usage experience. Many existing studies use the term willingness to pay (WTP), and these studies typically focus on estimating the actual monetary value of goods that are unpriceable. Willingness to pay in this study focuses on estimating support for costly polices rather than estimating their actual prices. In this regard, the willingness to pay literature in the marketing field is discussed at the strategic level and has focus on the exploration of attitudes [1]. Based on this willingness to pay research trends, this study explores the determinants of the general public's willingness to pay for PM and damage mitigation policies.

Fine dust pollution is a social problem that requires public participation and public payment to solve. PM is composed of heavy metals or toxic substances which cause diseases and social costs, and considerably impacts people's lives and health. PM was designated as a first-class carcinogen by the International Agency for Research on Cancer in 2013. A survey on the awareness of citizens regarding the air environment in Seoul confirmed that citizens are aware of PM problems and are willing to actively deal with them [2]. Based on the opinions suggested at the Seoul Citizens' PM Discussion Meeting (2017), the Seoul Metropolitan Government announced the Seoul Metropolitan Air Quality Improvement Plan for 10 projects; one suggestion was encouraging citizens to participate in the two-part vehicle system. The '“free public transportation policy” was also implemented. However, the policy was officially withdrawn after two months, amid criticism of its low efficiency vis-à-vis the cost of PM reduction [2]. This policy was implemented to induce public participation in a situation wherein the costs and benefits of the government policy and citizens' participation intentions were not accurately matched, suggesting the importance of understanding the intention to participate in advance.

Currently, most empirical studies on the subject focus on deriving a specific value for PM reduction or securing the validity of the policy through socioeconomic benefits. Additionally, the literature has focused on the factors affecting WTP. According to previous research, sociocultural background, personal experience, trust in policies and values, etc. influence an individual's WTP.

However, existing studies are limited in that WTP is often constructed as a single dimension. For example, Choi and Seok [3] found that WTP is used to estimate the value of a service based on the stated value or to evaluate its utility for non-market services. Na and Yoo [4] evaluated a target policy by measuring WTP. Empirical studies of WTP have been conducted in the areas of finance, personal information, products, and tourism [5,6]. These studies have considered a single dimension of WTP, thus have produced results that are limited to the dimension under study. Since WTP can be influenced by various variables, an integrated model considering all of them is needed [7].

Therefore, this study constructs a multidimensional, rather than unidimensional, approach to examine willingness to pay. This study divided willingness to pay into three categories: (1) cost burden, (2) benefit to community, and (3) benefit to specific groups. The reason for this division into three dimensions is that each dimension is an important indicator of willingness to pay and each dimension needs to be compared. Specifically, the meaning of each dimension is as follows.

First, the dimension of cost burden was considered because it is the most important factor that constitutes willingness to pay for fine dust reduction policies. Considering the overall influence of the factors that determine cost burden is important to understand the determinants of cost burden. It is also necessary before distinguishing the following two dimensions. This is because the next two dimensions separate benefits into distributive and redistributive policy, respectively.

Second, the willingness to pay for the community environmental improvements is aligned with the dimension of distributive policy. Therefore, looking at PM reduction policies as a benefit to the community has implications for estimating support for and willingness to pay for distributive policy. It is important to examine the willingness to pay dimension for projects that improve community conditions because perceptions of community may shape willingness to pay in different ways.

Third, considering the dimension of benefit to specific groups corresponds to a redistributive stance on PM reduction policies. The willingness to pay for benefit for specific groups shows the characteristics of groups that want more efficient and focused problem solving. Therefore, the distinction and comparison of these three dimensions can contribute to reducing resistance and increasing support for implementing PM reduction policies.

Based on the above, this study expects the determinants of WTP to be significantly different for each dimension. Thus, this study posits:

Hypothesis 1 **(H1**). The determinant structure of the three dimensions of WTP (benefit to the community, benefit to specific groups, and cost burden) will differ.

### 2.2 Different determinant factors for the multidimensionality of WTP

Few studies have focused on PM reduction behavior and the factors that influence these behavioral intentions [8,9]. This study assumes multidimensionality of WTP, which implies that there are different structures for determining the different dimensions. It can be assumed that the influence of the same variables on WTP will vary, depending on the target policy. To explore the influence of various factors that determine WTP, this study set three main factors as independent factors: resources, planned behavior, and government/policy. The reason for focusing on these particular factors is because they perform different functions. As they possess attributes distinct from each other, each of them has a domain that can be explained well. For individuals, resources are the objective conditions for action, whereas attitudes, norms, and feelings of control are subjective. While the resources and planned behavior factors are the primary variables that influence individuals as subjects of responsibility, government/policy factors are the variables that influence these subjects as objects that enable them to take action. These three main factors are more specifically described below.

First, the resource factors are required to act. Resources are objective assets that individuals possess, such as income, health, environment, and information. Without resources, goal-directed behavior is weak. This is the basic premise of payments for certain things. In risk research, under-resourced vulnerability affects people's behavior. Benford et al. [10] proposed the Vulnerability Hypothesis in which socially disadvantaged groups have higher risk perceptions because they lack the "resources and alternatives" to gain benefits and avoid disadvantages in decision-making related to defense. Klineberg et al. [11] shows that income is a resource that positively influences environmental behavior. Health is also a factor that raises environmental concerns and makes people responsive to PM reduction policies because the physical environment, such as air quality, is directly related to human survival and quality of life [12]. Royne et al. [13] confirms this through empirical analysis. Udalov & Welfens [14] analyze OECD countries and show that information from a variety of media influences environmental concerns and environmental action behaviors. In this study, resource factors consisted of income resources, personal health, environment/facility resources, and information, all of which are individuals can control.

Second, in Theory of Planned Behavior (TPB), planned behavior consists of attitudes, norms, and feelings of control related to an individual's subjective conditions for action [15]. TPB is a model wherein perceived control variables are added to the existing theory of reasoned action (TRA) [15]. TPB holds that the best predictor of individual behavior is intention, which is influenced by attitudes, norms, and feelings of control. Generally, the stronger the intention to engage in a behavior, the stronger its performance [15]. TPB is considered a self-interest theory; all its variables were rational choice predictors that regarded pro-environmental behavior as resulting from an individual's cost–benefit analysis [16]. Ajzen and Madden [17] showed experimentally that TPB demonstrates more accurate explanatory power for intentions and goal attainment than TRA. Son and Lee's meta-analysis [18] confirmed the assumption that attitudes toward people, subjective norms, and perceived behavioral control affect behavioral intentions. Park et al.’ study [19] shows that the greater the risk perception of particulate matter, the higher the behavioral intention. Perceived risk is associated with psychological discomfort and leads to increased avoidance behaviors. This, in turn, increases willingness to pay to reduce PM [20].

Third, the government is the actor, while policy is the means that this actor mobilizes to achieve a particular end. The government is the most important actor because it holds diverse policy tools for controlling PM. To solve PM related problems, the government can directly intervene through policies; however, this is often impossible without public participation. The public's attitudes and sentiments toward the government and its policies influence their behavior. This study adopted four government/policy variables as independent variables to explain the WTP level for PM reduction: trust in the government, policy satisfaction, policy preference, and policy knowledge.

This study expects the impact of each factor on WTP to differ, because of their different natures. In the following sections, this study derived hypotheses for the sub-variables in each resources, planned behavior, and government/policy factor.

Hypothesis 2 **(H2)**. Resources, planned behavior, and government/policy factors have different structural influences on the three willingness to pay dimensions.

### 2.3 Predictor 1: resource factor

#### 2.3.1 Income

Income is a primary sociodemographic variable and is generally used as a control variable. Income is the variable most directly related to WTP because it is a monetary resource. Low-income groups are primarily affected by PM but are less likely to respond because they lack the resources to do so. Choi and Suk [3] analyzed the WTP about the policy opposition to public safety service-related policy target groups, such as emergency medical care, traffic accidents, and PM. They reveal that “both the lack of ability of households to pay” and “distrust in the will of the government,” were the main causes of rejection. In Park and Kim's study [21], high household income was a factor driving PM response behavior. Therefore, the household income level was added as a predictor of WTP , and this study posits.

Hypothesis 3 **(H3)**. The higher the household income, the higher the WTP for PM reduction.

#### 2.3.2 Health

Health status is affected by PM. The more affected one is by PM, the worse one's health is. If health deteriorates because of PM, response behavior and WTP will naturally increase. In Kim's study [22], WTP was measured through health-related variables such as experience of disease symptoms, number of days of restriction, avoidance behavior costs, mitigation behavior costs, and number of chronic disease experiences. Mitigation behavior, which is intended to improve health, had a significant positive effect on WTP. Conversely, Eom et al. [23] noted that avoidance behavior costs had the largest impact. When PM exposure is likely to cause a health risk, active avoidance behaviors can effectively reduce social costs. However, this pattern depends on the individual's subjective perception of risk; thus, this study posits:

Hypothesis 4 **(H4)**. The lower the health resource, the higher the WTP for PM reduction.

#### 2.3.3 Facility

This study assumed that WTP would vary depending on the facility ownership status that enables individuals to cope with PM damage. Responding to PM damage requires various physical facilities and devices, including purifiers, sterilizers, and PM meters, provided in homes and main activity spaces to reduce its negative impact. The perception of comfort level in the residential area under PM threat was related to vehicular traffic, industrial facilities, aged buildings, and the presence of polluting businesses. Appropriate facilities encourage active behavior to reduce PM and increase the willingness to pay for PM.

Hypothesis 5 **(H5)**. The more facility resources people have, the higher their WTP for PM reduction.

#### 2.3.4 Information

Decisions about an individual's behavior are not made in a vacuum but are based on information. The amount and content of information determine the direction of decisions. Research on information has focused on its role and effects, whether positive or negative, and the amount of information the public possesses. Beck [24] found that negative information influences an individual's beliefs and attitudes, while positive information influences behavioral intention. Kim et al. [25] found that the amount of information, rather than content, influences perceptions of nuclear accidents and nuclear risk. The higher the level of use of information resources related to PM, the more active for PM reduction. Thus, this study posits:

Hypothesis 6 **(H6)**. The higher the level of information resource use, the higher the WTP for PM reduction.

### 2.4 Predictor 2: planned behavioral factors

#### 2.4.1 Attitude

TPB stresses on the relationship between attitude/norms and intention/action. Attitude is defined as a positive or negative evaluative opinion of something. According to Ajzen and Fishbein [26], these intentions are influenced by two factors: (a) attitude toward the act in question, and (b) perceived normative expectations from reference groups, multiplied by the motivation of a person to comply with expectations. Focusing on the subjective perception of risk, Fischhoff et al. [27] and Slovic et al. [28] proposed a psychometric paradigm that implies the perceived risk and benefit of an object vary subjectively from person to person. Perceived risk is the perceived level of danger from a hazardous object [29]. Generally, the more people perceive PM as dangerous, the more they perceive the benefits of addressing it, and the more likely they are to act. The most basic idea regarding PM is the risk involved and the benefits of addressing it. Park and Kim [21] found that both, perceived risk and benefit had a positive effect on fine dust response behavior. Thus, this study posits:

Hypothesis 7 **(H7)**. The higher the perceived risk, the higher the WTP for PM reduction.

#### 2.4.2 Norms

Norms are invisible external moral burdens imposed on individuals. Ajzen and Fishbein [30] demonstrated that not only attitudes toward acts, but also related normative beliefs, influenced behavioral intentions for acts pertaining to one, or dichotomous and multiple-choice situations. Similarly, Ajzen and Fishbein [26] showed that both attitudes and normative beliefs about behaviors are highly related to intentions to perform those behaviors. Moreover, in TPB, the perceived locus of control can explain the intention to act. Ajzen [15] explained that intentions to perform different types of behaviors can be predicted with high accuracy from attitudes toward the behavior, subjective norms, and perceived behavioral control. Norms are categorized as personal or social. Personal or subjective norms refer to internal moral pressures, while social or descriptive norms refer to external collective pressures. In urgent PM reduction demands, social norms for individuals may be more persuasive (Moan and Rise, 2011). According to Shi et al. [31], perceived behavioral control and social norms significantly affect PM reduction intention. Thus, this study posits:

Hypothesis 8 **(H8)**. The greater the personal norms, the higher the WTP for PM reduction.

Hypothesis 9 **(H9)**. The greater the social norms, the higher the WTP for PM reduction.

#### 2.4.3 Perceived control

The perceived behavioral control variable was added to the first TRA model [32] and is defined as the perceived ease or difficulty of performing a particular behavior [15]. This is a type of self-efficacy regarding the locus of control. According to Shi et al. [31], perceived behavioral control directly and significantly affects PM2.5-reduction intention and moderates the relationship between household moral norms and PM2.5-reduction intention. The more ease a household thinks, the more likely it is for it to engage in PM reduction behavior. If people perceive difficulty in commuting by public transportation, for example, they will hesitate to do something to reduce PM [31]. Thus, this study posits:

Hypothesis 10 **(H10)**. The higher the perceived sense of control, the higher the WTP for PM reduction.

### 2.5 Predictor 3: government and policy factors

#### 2.5.1 Trust in government

Trust refers to the degree of credibility of an object, attribute, or process. In many cases, the focus has been on trust in actors [21]. Park and Kim [21] found that higher trust in the government and lower trust in rumors were associated with higher PM-related coping behaviors. Not only attitudes, norms, and emotions, but also trust in government plays a role in increasing public acceptance towards low-emission zones. Trust in government supports beliefs about low-emission zones [33]. Shin et al.' s research [34] shows that citizens' positive attitudes toward government and confidence in government increase individual responsiveness. This increases PM reduction behavior. Kim and Moon [35] found that when trust in government is high, individuals have a strong belief that the government will protect them in times of danger, making them more compliant with policies. These findings suggest that high trust in government is associated with positive support for policies to reduce PM. Thus, this study posits:

Hypothesis 11 **(H11)**. The higher the trust in government, the higher the WTP for PM reduction.

#### 2.5.2 Policy satisfaction

From the utilitarian perspective, satisfaction generates positive attitude. Satisfaction with the government drives support for relevant policies. Yoo and Moon's study [5] on the determinants of attitudes toward tax increases to respond to climate change observed that taxpayers will exhibit a positive attitude toward tax payment if they believe that the public services provided by the government are worthwhile. Cho et al. [36] revealed satisfaction with government performance was a component of government outcomes. However, there are some oppositions to pay taxes [37,38]. Further, empirical studies show that the evaluation of policy effectiveness is an important determinant of attitude toward tax increases [39,40], revealing that satisfaction and trust in policies have a positive effect on tax increases. Thus, this study posits:

Hypothesis 12 **(H12)**. The higher the satisfaction with government policy, the higher the WTP for PM reduction.

#### 2.5.3 Policy preference

Policy preference denotes a system of priorities for a policy or policy alternative from the perspective of an evaluation. People evaluating policies will show high trust in government if it pursues their preferred policy [29]. Consequently, a positive evaluation of a policy induces responsive behavior toward PM. A number of recent studies have identified public support as an essential factor for increasing the effectiveness of climate change mitigation policies, and in particular, public preferences for policies are an important mechanism for increasing policy effectiveness [41,42]. Stadelmann-Steffen & Eder [43] focus on policy preferences in a study of European countries and find that policy preferences and continuous policy feedback from the government play an important role in citizens' support or opposition to policies. Furthermore, they show that preferences for existing policies influence support for new policies. Thus, this study posits.

Hypothesis 13 **(H13)**. The higher the preference (urgency) of a PM-related policy, the higher the WTP for PM reduction.

#### 2.5.4 Policy knowledge

Shi et al. [8,9] demonstrated the power of knowledge: more knowledge about climate change is a driver of public supports. Moreover, knowledge positively influences climate change policy acceptance [8]. Park and Kim [44] found that both objective and subjective knowledges have individual influences on nuclear acceptance. In particular, subjective knowledge had a stronger influence on the acceptance of nuclear power at the individual level, whereas only objective knowledge affected acceptance of nuclear power at the social level. A study by Stoutenborough et al. [45] also shows that knowledge affects support for nuclear energy policy. Knowledge is one of the most important factors in the problem-solving process, and when individuals have inaccurate information, it is difficult for them to choose an appropriate solution [46]. Especially when the issue is complex or involves uncertainty, individuals tend to utilize even inaccurate information and rely on more accurate information for issues that have a lasting impact on human survival and prosperity, such as environmental issues [47]. Therefore, this study hypothesizes that the willingness to pay for PM reduction policies will be influenced by policy knowledge.

Hypothesis 14 **(H14)**. The higher the level of knowledge on PM policy, the higher the WTP for PM reduction.

## 3 Materials and methods

### 3.1 Data collection

The data used in this study was collected from the Fine Dust Information Center's "2021 National Emission and Air Quality Assessment System Preparation Study II". The survey was conducted from February 21 to 27, 2022, using a professional survey company in Korea. The survey was conducted among 500 members of the general public. The sample size was based on the Korean Ministry of the Interior and Safety's resident registration population statistics (as of September 2021) and was proportionally allocated according to region, age, and gender. The research method was to build a questionnaire and conduct the survey online (web & mobile) (CAWI: Computer Aided Web Interviewing & CAMI: Computer Assisted Mobile Interview). The questionnaire consisted of about 282 questions. In the process of executing the online response system, a logic was built to prevent missing questionnaires and insincere responses.

The sample consisted of adult men and women aged 19 and older in South Korea. The sample design was based on demographics, and it was allocated by proportional representation, as shown in the following equation.

$$n_{h}=n\frac{N_{h}}{\sum_{k=1}^{k}\sum_{i=1}^{i}\sum_{j=1}^{j}N_{kij}}$$

* $N_{h}$ is the number of survey subjects in the population of h.

∗ n: Sample size, k: Region, i: Age group, j: Gender (male/female)

To ensure the accuracy of the survey, this study simulated two options (see below option 1 and option 2) and compared each plan to select the initial sample design. Option 1 had the advantage of relatively low cost and quick data collection. However, in the end, this study chose the second option because it allowed for accurate regional representation.

Option 1: 7 regions, gender, age.

Option 2: 16 cities, gender, age.

Looking at the characteristics of the sample, 246 participants (49.2 %) were male and 254 (50.8 %) were female; 83 people in their 20s (16.6 %), 76 in their 30s (15.2 %), 92 in their 40s (18.4 %), 99 in their 50s (19.8 %), and 150 in their 60s (30.0 %). Regarding educational background, 114 people (22.8 %) had a high school graduate or less, and 386 people (77.2 %) had college graduates or higher. Because the standard for proportional distribution was set by gender, age, and region, without considering academic background, the proportion was high with 317 (63.4 %) college graduates or higher.

### 3.2 Measurement items and reliability analysis

The dependent variables were categorized as: WTP for cost burden, WTP for benefit to the community, and WTP for benefit to specific groups. Independent variables were classified into three factors: resources, planned behavior, and government/policy factors, and each factor consisted of four measurement concepts.

For the measurement method, a 5-point Likert scale was used for all resource factors except for income and facility resources. Most of the questions were asked for the degree of agreement, with 1 being strongly disagree and 5 being strongly agree. Policy Knowledge were asked how much they knew about each fact on a 5-point scale. Income resources were analyzed using a log function after measuring it with open question, and the size of facility resources was calculated by measuring the presence or absence of facility possession and then summing of numbers of facility resources.(see. Table 1)

**Table 1:** Measurement items and reliability analysis results.

Division	Measurement concept	Statement		Cronbach α
**Independence 1: Resource Factors**	**Income resource**	What is your overall average monthly gross income?		–
	**Health status**	I am healthy.		0.851
		I have a healthy body compared to other people.		
	**Facility resources**	Presence of residential housing and main activity space (home, activity space)	Air purifier	–
			Air sterilizer	
			PM meter	
			Ventilator	
			bathroom exhaust fan	
	**Information**	Citizen/environmental groups or NGOs (Non-Governmental Organizations)		0.769
		Offline media (broadcasting, paper newspapers and magazines, etc.)		
		Online press (Internet newspaper, portal news, etc.)		
		Personal blogs, SNS, cafes, and communities		
		Applications (fine particles, air pollution information, Air Korea, etc.)		
		Neighbors (friends, family, etc.)		
**Independent 2: Planned behavior factors**	**Perceived risk**	Diseases caused by PM have very serious consequences.		0.740
		Illness caused by PM will have a huge impact on my life.		
		When PM is resolved, our society will develop significantly.		
	**Personal norms**	I believe that the reduction of PM is absolutely necessary for the next generation.		0.791
		I believe that nationwide efforts are needed to reduce PM.		
	**Social norm**	People who are important to me say that I should participate in PM reduction actions.		0.771
		People who matter to me expect me to participate in PM reduction action.		
	**Perceived sense of control**	I can participate in PM reduction actions if necessary.		0.720
		I have sufficient capacity to participate in PM reduction actions.		
**Independence 3:Government/Policy Factors**	**Trust in government**	Government		0.700
		Environment-related specialized institutions (Ministry of Environment, National Institute of Environmental Science, Korea Environment Corporation, PM Information Center, Korea Meteorological Administration)		
		The government should be responsible for solving PM rather than individuals.		
	**Satisfaction**	I am satisfied with PM reduction policy that the government is promoting.		0.824
		I am satisfied with the PM policy countermeasures.		
	**Policy preference**	PM problem is the most urgent problem to be solved.		0.694
		PM is the most important policy agenda to be addressed in the country.		
	**Policy knowledge**	PM seasonal management system (preventive special measures to intensively manage PM by operating stronger reduction measures than usual from December to March of the following year.)		0.827
		Only vehicles with odd (even) digit at the end of the vehicle number can be operated on odd (even) days for national and public institutions located in the metropolitan area and 6 special and metropolitan areas.		
		We are providing financial support for replacement of eco-friendly boilers in old household boilers.		
		The Seoul Metropolitan Government will pay mileage for citizens who drive less than 1850 km during PM season management system (December to March of the following year).		
		We are expanding the supply of LPG vehicles and eco-friendly vehicles.		
		Vehicle emission grade 5 is restricted from driving due to the large amounts of pollutants emitted.		
		We support the installation of air purification facilities in public transportation vehicles.		
		Distribute health masks to sensitive and vulnerable populations.		
**Willingness to pay**	**Willingness to pay for cost burden**	Carbon tax		0.855
		Electricity price increase		
		Raising money for PM reduction		
		Government raises money		
		An environmental organization raises money		
	**Willingness to pay for benefit to community**	Creating a forest within a region/village		0.848
		Supply of eco-friendly boilers		
		Supply of eco-friendly vehicles		
		Energy conversion from fossil fuel power generation to renewable energy		
		Installation of air purification facilities in schools, daycare centers, etc.		
	**Willingness to pay for benefitto specific groups**	Blocking PM for the underprivileged/Expansion of ventilation facilities		0.762
		Masks and air purifiers for the underprivileged		
		Financial support for PM reduction facilities for companies		

## 4 Results

### 4.1 Comparative analysis by group

**Fig. 1:**
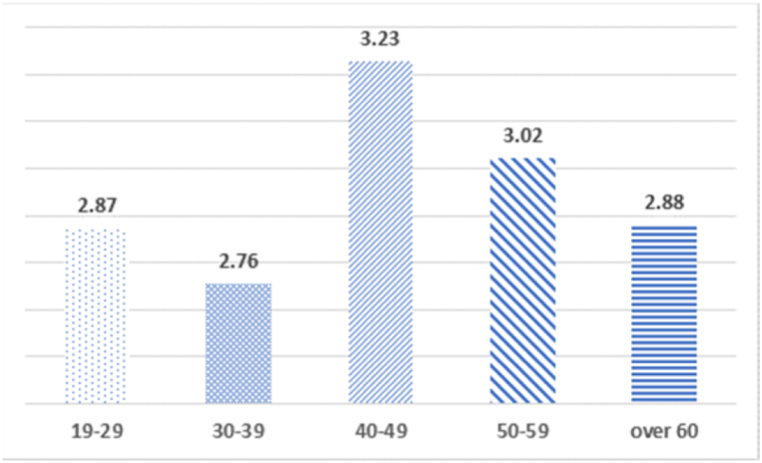
Analysis of the average difference between age and willingness to pay for cost-burden.

**Fig. 2:**
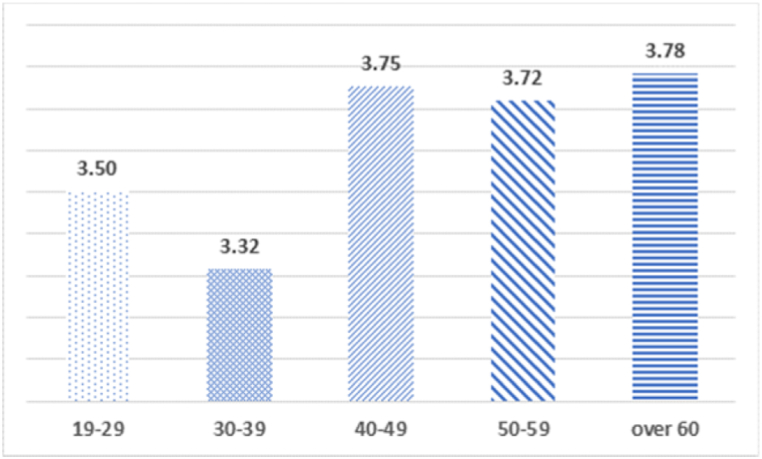
Analysis of the average difference between age and willingness to pay for benefit to the community.

**Fig. 3:**
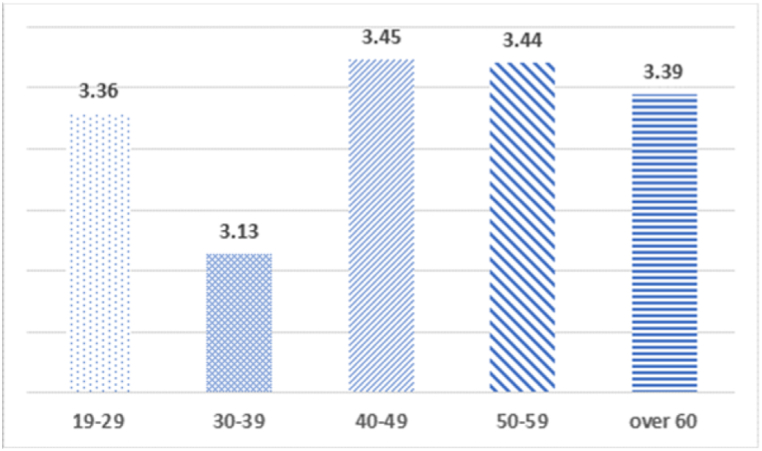
Analysis of the average difference between age and willingness to pay for benefit to specific groups. *p < 0.05, **p < 0.01, ***p < 0.001.

A comparative analysis of the mean between groups of control variables for the three types of WTP for PM reduction was performed. As a result of T-test and one-way ANOVA, by inputting gender, age, academic background, and political ideology, there were no significant average differences in gender, academic background, and political ideology. The average differences by age are shown in Fig. 1, Fig. 2, Fig. 3. Significant average differences were found between aged groups in WTP for benefit to the community and WTP for cost burden. The Scheffé test was conducted to identify the group which demonstrated differences. The results confirmed significant average difference between those in their 30s and 40s (p <00.01), and between those in their 40s and 60s (p <00.05) in case of cost-burden model. In terms of willingness to pay for benefit to community, significant mean differences were confirmed among those in their 30s and 40s (p <00.01), 30s and 50s (p <00.05), and 30s and 60s (p <00.001).

### 4.2 Regression

Linear regression analysis was performed to identify the variables and causal structures affecting the WTP for PM reduction. After checking the prerequisites for regression analysis such as multicollinearity, the variance inflation factor (VIF) values ranged from a minimum of 1.088 to a maximum of 1.911. When the VIF is 10 or greater, it is a criterion for judging whether there is multicollinearity, and there are cases where the criterion is more strictly set to 5. However, in this analysis, the result of the collinearity diagnosis was 1.911, which excluded the possibility of multicollinearity.

In Table 2, Model 1 employed traditional WTP for cost burden as a dependent variable, and confirmed that political ideology, facility resources, information resources, social norms, perceived control, and policy satisfaction increase WTP. The more progressive the political ideology, the higher the levels of facility resource ownership, information resource use, satisfaction with the government's PM reduction policy and the higher the recognition of social norms or perceived control, the higher the willingness to pay for cost burden (see Table 2and Table 3).

**Table 2:** Verification of mean difference between independent and dependent variable groups.

			Willingness to pay for cost burden		Willingness to pay for benefit to community		Willingness to pay for benefit to specific groups	
			medium	t-value	medium	t-value	medium	t-value
**Resource factors**	**Income**	below average	2.826	−2.861 **	3.520	−3.243 **	3.233	−3.221 **
		above average	3.040		3.738		3.457	
	**Health status**	below average	2.822	−4.126 ***	3.547	−3.466 **	3.253	−3.724 ***
		above average	3.119		3.778		3.509	
	**Facility**	below average	2.846	−3.506 ***	3.569	−2.813 **	3.271	−3.271 **
		above average	3.102		3.759		3.498	
		above average	2.792		3.713		3.319	
	**Information**	below average	2.715	−6.579 ***	3.533	−3.351 **	3.169	−5.639 ***
		above average	3.174		3.755		3.548	
**Planned behaviorfactors**	**Perceived risk**	below average	2.878	−1.999 *	3.461	−5.623 ***	3.264	−2.875 **
		above average	3.022		3.826		3.461	
		above average	3.050		3.826		3.473	
	**Personal norms**	below average	2.873	−2.733 **	3.463	−7.672 ***	3.287	−2.979 **
		above average	3.089		3.967		3.499	
	**Social norms**	below average	2.631	−6.470 ***	3.565	−1.744	3.157	−4.319 ***
		above average	3.111		3.689		3.468	
	**Perceived sense of control**	below average	2.768	−5.477 ***	3.426	−7.392 ***	3.160	−6.558 ***
		above average	3.158		3.897		3.596	
**Government/policy factors**	Trust in g**overnment**	below average	2.724	−4.946 ***	3.406	−5.724 ***	3.204	−3.615 ***
		above average	3.087		3.790		3.460	
		above average	2.931		3.777		3.422	
	**Policy satisfaction**	below average	2.594	−7.414 ***	3.549	−2.077 *	3.146	−4.807 ***
		above average	3.149		3.702		3.486	
	**Policy preference**	below average	2.829	−3.311 **	3.367	−8.683 ***	3.184	−5.174 ***
		above average	3.066		3.909		3.533	
	**Policy knowledge**	below average	2.739	−6.453 ***	3.457	−6.261 ***	3.178	−5.904 ***
		above average	3.190		3.861		3.573

**Table 3:** Regression analysis results.

Category		Model 1 Willingness to pay for cost burden			Model 2 Willingness to pay for benefit to community			Model 3 Willingness to pay for benefit to specific groups		
		b	SE	Beta	b	SE	Beta	b	SE	Beta
(Constant)		−0.254	0.361		0.313	0.355		0.542	0.375	
Control variable	Gender	−0.012	0.061	−0.007	−0.028	0.060	−0.019	−0.074	0.063	−0.048
	Age	−0.001	0.002	−0.015	0.005*	0.002	0.099	−0.001	0.002	−0.016
	Education	0.021	0.076	0.011	0.056	0.074	0.031	−0.109	0.078	−0.059
	Political ideology	0.048**	0.018	0.097	0.017	0.018	0.037	0.007	0.019	0.016
Independent variable 1: Resource Factors	Income	−0.012	0.043	−0.011	0.011	0.042	0.011	0.071	0.045	0.069
	Health status	0.030	0.039	0.029	0.046	0.038	0.049	0.033	0.041	0.034
	Facility	0.033*	0.016	0.084	0.031*	0.015	0.085	0.027	0.016	0.073
	Information	0.174***	0.046	0.165	0.030	0.045	0.031	0.063	0.048	0.063
Independent variable 2: Planned behavior Factors	Perceived risk	0.042	0.048	0.037	0.069	0.047	0.066	0.026	0.049	0.024
	Personal norms	−0.013	0.053	−0.012	0.135*	0.052	0.137	−0.008	0.055	−0.008
	Social norms	0.196***	0.042	0.198	0.006	0.041	0.006	0.116**	0.044	0.123
	Perceived sense of control	0.109*	0.055	0.092	0.101	0.054	0.093	0.131*	0.057	0.117
Independent variable 3: Government/Policy Factors	Trust in government	0.071	0.041	0.073	0.097*	0.040	0.108	0.025	0.043	0.027
	Policy satisfaction	0.256***	0.043	0.256	0.026	0.042	0.028	0.101*	0.045	0.106
	Policy preference	0.020	0.049	0.018	0.154**	0.048	0.152	0.131**	0.050	0.125
	Policy knowledge	0.061	0.053	0.054	0.116*	0.052	0.109	0.184***	0.055	0.169
F value		18.195***			12.425***			10.545***		
adj.R^2^		0.355			0.268			0.234		

Note:*p < 0.05;**p < 0.01; and.***p < 0.001.

In this model, the WTP for cost burden includes an ideological issue. Progressives differ from conservatives in those progressives prefers the burden of costs of government intervention, while conservatives emphasize the minimal intervention and cost burden. This traditional ideology plays a role in PM.

Second, facility resources and information resources play a significant role in resource factors. Interestingly, income and health resources, which have a direct relationship with individuals, are insignificant, while environmental and information factors play a significant role. These results imply that since PM is a macroscopic problem that individuals cannot control. Therefore, responses should be made at the social and national levels rather than at the individual level. Consequently, infrastructure such as facility resources plays an important role.

Third, social norms and perceived sense of control are significant in inducing individuals' willingness to pay. This suggests that to improve willingness to pay for PM policy, a sense of moral obligation towards the community should be triggered through external pressure at the collective level. The significance of “perceived sense of control” indicates that individuals' sense of control or confidence in policy issues plays an important role, suggesting that citizens' sense of authority should be strengthened, and citizenship be cultivated by increasing citizen participation in policy.

Fourth, policy satisfaction is the only significant variable among the government and policy factors. To induce a consumer's payment action from the point of view of demand in the market, utility must be maximized through the supplied product. The significance of policy satisfaction suggests that this general logic can also be applied to policy issues. Among the variables influencing WTP, policy satisfaction was the largest, followed by social norms and information resources, and the explanatory power of the model was 35.6 %. The result showing an explanatory power of high satisfaction suggests that policy efforts to maximize individual utility are required to induce a cost burden.

In Model 2, the WTP for benefit to the community is a dependent variable, and variables with a significant causal relationship between resource factors, planned behavioral factors and government/policy factors were identified. First, in the resource factor, it was confirmed that the higher the facility resources, the higher the WTP for the community environment improvement. In addition, unlike Model 1, personal norm variables showed a significant influence in planned behavior factors, and in policy factors, WTP for the community environment improvement increased as trust in government, policy preference, and policy knowledge increased.

Based on the standardized regression coefficient values, the size of the influence of the independent variables appeared in the order of policy preference, personal norms, policy knowledge, trust in government, and facility resources, and the explanatory power of the model was 26.8 %.

What is noteworthy in Model 2 is that the TPB and policy factor variables appeared different from the significant variables in Model 1. In Model 1, social norms and perceived control were significant, and the personal norm variable was not significant. However, in Model 2, only personal norm variables were found to affect community WTP. This result is interpreted as the fact that the WTP for improvement of the community environment differs from WTP for the traditional cost burden. Since the benefit of community projects are experienced by the community rather than the individual, individuals with selfish motives will not be inclined to pay. However, in the case of those with a sense of moral obligation in the normative dimension, there will be a certain commitment to community-related businesses, regardless of individual benefits. Therefore, in this study personal norms appeared to be significant variables in the TPB.

Government and policy factors also have an impact. As PM is a macro-environmental problem, it cannot be solved by individual efforts alone, and government and policy tools must also play an important role. In this respect, the fact that trust in government, policy preference, and policy knowledge are significant confirms the importance of government and policy. In particular, when looking at the standardized coefficient values, policy preferences have the greatest explanatory power, followed by policy knowledge. These results confirm the importance of policies in relation to cost payments. Second, policy knowledge also being important suggests that information on PM related policies, currently promoted by the government, should be disseminated more actively. Third, trust in the government being significant suggests that the government's role in deciding who will operate PM-related projects is vital.

Model 3 shows the WTP for specific groups as a dependent variable. In Model 3, unlike Models 1 and 2, no significant variables appeared in the resource factors. Policy knowledge had the greatest influence on WTP for support of a specific groups, followed by perceived control, policy preference, social norms, and policy satisfaction. The explanatory power of Model 3 was found to be 23.4 %.

Model 3 differs from Models 1 and 2 in resource factors. Because support for specific groups has a redistributive nature, individual resistance appears relatively weak compared to WTP for the community and cost burden. Meanwhile, in the TPB factor, the WTP for a specific groups shows a similar form to the WTP for cost burden, and the fact that it is different from WTP for the community is a noteworthy result. Considering that the specific groups in this study is a vulnerable group, this can be interpreted as because WTP for them follows social norms and a sense of control rather than individual ones.

Similarly, regarding policy factors, individuals with higher policy knowledge have lower sensitivity to risks related to damage and PM reduction, which is not perceived as a major risk. Ultimately, this can be interpreted as leading to the support of a small group of people, not the individual. In the same context, policy preference, which refers to satisfaction with and urgency of policy, rather than individual benefits or norms, affects the WTP for support for a specific groups.

## 5 Discussion

This study aimed to confirm the multidimensionality of the WTP for PM reduction and explore the factors that determine it. To this end, resource, planned behavior, and government/policy factors were established through a review of domestic and foreign research, and the WTP for PM reduction was analyzed by classifying these factors into cost burden, benefit to the community, and benefit to specific groups. Analysis results are as follows:

In Table 4, this study confirmed fully hypothesis 1 (the determinant structure of the three dimensions of WTP (benefit to the community, benefit to specific groups, and cost burden) will differ). As you can see in Table 4, the determinant structure for the three willingness to pay groups is changing. Also, hypothesis 2 (resources, planned behavior, and government/policy factors have different structural influences on the three willingness to pay dimensions) is confirmed. Also, facility resources (Hypothesis 5) and information resources (Hypothesis 6), personal norms (Hypothesis 8), social norms (Hypothesis 9 ), perceived control (hypothesis 10), trust in government (hypothesis 11), policy satisfaction (hypothesis 12), policy preference (hypothesis 13) and policy knowledge (hypothesis 14) were confirmed to have an effect on the one of three WTPs. In contrast, income (hypothesis 3), health (hypothesis 4), and perceived risk (hypothesis 7) did not affect any ones of WTPs. This is a different result from the general rule that the higher the income or health, the higher the WTP.

**Table 4:** Hypothesis test results.

Division	Measurement concept	Hypothesis	1. Willingness to pay for cost burden	2. Willingness to pay for benefit to community	3. Willingness to pay for benefit to specific groups
**Independence 1: Resource factors**	H3**: Income**	+			
	H4**: Health**	–			
	H5**: Facility**	+	+	+	
	H6**: Information**	+	+		
**Independent 2: Planned behavior factors**	H7**: Perceived risk**	+			
	H8**: Personal norms**	+		+	
	H9**: Social norms**	+	+		+
	H10**: Perceived sense of control**	+	+		+
**Independence 3:Government/Policy factors**	H11**:Trust in government**	+		+	
	H12**: Policy satisfaction**	+	+		+
	H13**: Policy preference**	+		+	+
	H14**: Policy knowledge**	+		+	+

The analysis revealed no independent variable with consistently significant effects on the three dependent variables. This suggests that the same variable has different effects depending on the different dimensions of WTP for PM reduction. In terms of resource factors, facility and information resources had a statistically significant effect on the burden of costs, such as carbon tax, electricity rate increase, and cost collection, but did not affect the cost for the community environment improvement, or for support from the underprivileged or companies. Among the planned behavior factors, personal norms had a significant effect only on community WTP, while social norms and perceived control had an impact on cost burden WTP and specific groups WTP. Trust in government had an effect only on WTP for the community, and policy satisfaction had an effect on WTP for cost burden and WTP for specific groups. Policy preferences and policy knowledge influenced WTP for the community and WTP for specific groups.

As a result of comparing the results of the regression analysis, it was found that the statistical influence of the independent variables was comparable to that of information resources, policy preference, and policy knowledge. Additionally, personal norms and trust in government were contrasted with social norms, perceived control, and policy satisfaction. However, linear regression analysis has a limitation in that it is difficult to explain the contrast between the three types of WTP and independent variables.

The theoretical implication of this study is that even if the variables constituting TPB, the most representative theory explaining human intentions to act, are willing to pay for PM reduction, the contrasting effects depend on the nature of the policy. Even if the policies for the purpose of reducing PM are similar, the variables that determine the WTP vary if the beneficiaries or targets of the policies differ. Further, the behavior of the general public regarding personal and social norms must be determined before major premises such as PM reduction. Similarly, the effect on the WTP differs depending on whether the policy is distributed (the minority supports the majority) or redistributed (the majority supports the minority).

Second, this study distinguishes between willingness to pay for distributive policies (WTP for the community) and redistributive policies (WTP for specific groups) from a theoretical perspective and compares the influence of each variable. While policy knowledge is the most influential factor in the willingness to pay for specific group, the policy preference variable is the most important factor in the willingness to pay for the community environmental improvement. This is significant in that it empirically confirms that the mechanisms that determine willingness to pay are different depending on the nature of the policy, and in particular, the cognitive dimension of policy knowledge and the dimension of policy preference have different effects. According to a study by Kim [48], policy awareness and policy preference are distinct, and high preference may not mean high awareness. This study shows that the higher the policy knowledge, i.e., the higher the awareness of the policy, the higher the willingness to pay for the redistributive policy; the higher the preference for the policy, the higher the willingness to pay for the distributional policy. These findings are important in that they provide a basis for deciding whether to focus more on publicity and education to increase policy knowledge or on enhancing various positive images to increase favorability when designing future distributional and redistributive policies.

## 6 Conclusion

This study aimed to explore the determinants of the WTP for PM reduction. For this purpose, the WTP was examined by classifying it into the dimensions of cost burden, benefit to the community, and benefit to specific groups. The WTP in Model 2 (i.e., distribution-oriented WTP) is different from that in Model 3 (i.e., redistribution-oriented WTP). This suggests that the determinants of the public's WTP for a policy to reduce PM differ depending on the nature of the policy. Based on this, future PM reduction policies should be established considering the target of the policy and factors determining the payment decision.

In particular, for willingness to pay for specific groups, this study finds that policy knowledge, policy preferences, and interest in the policy itself, including policy satisfaction, are important influencers. This suggests that supportive policies for minorities or specific groups, such as redistributive policies, need to be carefully designed and actively promoted by policymakers. Even for policies that do not directly affect the individual, willingness to pay is related to prosocial behavioral intentions, which is also confirmed by regression results showing the impact of social norms and feelings of control. On the other hand, in the case of willingness to pay for the community development, which can be considered as distributive policies, trust in government is one of the main influencing variables, while policy preferences and policy knowledge also play a role. However, it is worth pointing out that trust in policy does not affect WTP to support specific groups. This suggests that individuals, as the ultimate beneficiaries, are more sensitive to government competence when it comes to distributional policies that directly affect them. As a result, individuals' trust in the government's ability to implement policies affects their final willingness to pay, and it is in the same vein that personal norms do not affect willingness to pay for specific groups, but only for the community. Different types of policies have different determinants of willingness to pay, and policy design needs to take this into account.

Limitations of this study include first, we only tested three of the many dimensions of willingness to pay, and more dimensions are needed. Second, the three independent variables are only one of many theoretical factors that need to be tested. Third, the composition of the 12 variables that make up the three factors relied on the author's arbitrary judgment and needs to be improved in the future.

## Formatting of funding sources

This work was supported by Korea CCUS Association(K-CCUS) grant funded by the Korea Government(MOE, 10.13039/501100003052MOTIE) (KCCUS20220001, Human Resources Program for Reduction of greenhouse gases). The 10.13039/501100002701Ministry of Education of the Republic of Korea and the 10.13039/501100003725National Research Foundation of Korea (NRF-2021S1A5C2A02087244).

## Disclosure instructions

The authors declare that they have no known competing financial interests or personal relationships that could have appeared to influence the work reported in this paper.

## CRediT authorship contribution statement

**Sehyeok Jeon:** Formal analysis, Data curation, Conceptualization. **Seoyong Kim:** Methodology, Investigation, Funding acquisition. **Sohee Kim:** Writing – review & editing, Visualization, Validation.

## Declaration of competing interest

The authors declare the following financial interests/personal relationships which may be considered as potential competing interests:Seoyong Kim reports financial support was provided by The Ministry of Education of the Republic of Korea and the National Research Foundation of Korea. Sohee Kim and Sehyeok Jeon reports financial support was provided by The Ministry of Education of the Republic of Korea and the National Research Foundation of Korea.

## References

[1] Kim S., Kim H., Kim N. (2020). Is the willingness to pay for public service a normative judgment on publicness or an empirical choice for public service?. Korean Public Administration Review.

[2] Kim H., Lee J. (2019). Why did seoul's free public transportation Program for reducing fine particulate matter fail?: a political management perspective. The Korean Journal of Local Government Studies.

[3] Choi H., Suk H. (2018). Discovering public service preferences of citizen groups using willingness to pay. The Korea Local Administration Review.

[4] Na H., Yoo C. (2019). Valuation of cultural policy through the contingent valuation method, using korea's ‘culture day’ as a case study. The Journal of Cultural Policy.

[5] Yoo N., Moon S. (2020). Who will agree with tax increase for tackling climate change?. Effects of Policy Satisfaction and Trust in Government. Modern Society and Public Administration.

[6] Cha K. (2015). Analysis of willingness to pay for financial counseling services. Consumer Policy and Education Review.

[7] Kim S., Kim S. (2023). Willingness to pay for what? Testing the impact of four factors on willingness to pay for facilitating and sanctioning energy policy instruments. Energy Rep..

[8] Shi J., Visschers V.H.M., Siegrist M. (2015). Public perception of climate change: the importance of knowledge and cultural worldviews. Risk Anal..

[9] Shi J., Visschers V.H., Siegrist M., Arvai J. (2016). Knowledge as a driver of public perceptions about climate change reassessed. Nat. Clim. Change.

[10] Benford R.D., Moore H.A., Williams Jr J.A. (1993). In whose backyard?: concern about siting a nuclear waste facility. Socio. Inq..

[11] Klineberg S.L., McKeever M., Rothenbach B. (1998). Demographic predictors of environmental concern: it does make a difference how it's measured. Soc. Sci. Q..

[12] Zimmer M.R., Stafford T.F., Stafford M.R. (1994). Green issues: dimensions of environmental concern. J. Bus. Res..

[13] Royne M.B., Levy M., Martinez J. (2011). The public health implications of consumers' environmental concern and their willingness to pay for an eco‐friendly product. J. Consum. Aff..

[14] Udalov V., Welfens P.J. (2021). Digital and competing information sources: impact on environmental concern and prospects for international policy cooperation. Int. Econ. Econ. Pol..

[15] Ajzen I. (1991). The theory of planned behavior. Organ. Behav. Hum. Decis. Process..

[16] Abrahamse W., Steg L., Gifford R., Vlek C. (2009). Factors influencing car use for commuting and the intention to reduce it: a question of self-interest or morality?. Transport. Res. F Traffic Psychol. Behav..

[17] Ajzen I., Madden T.J. (1986). Prediction of goal-directed behavior: attitudes, intentions, and perceived behavioral control. J. Exp. Soc. Psychol..

[18] Sohn Y., Lee B. (2012). An efficacy of social cognitive behavior model based on the theory of planned behavior: a meta-analytic review. Korean Journal of Journalism & Communication Studies.

[19] Park J., Park Y., Yoo J.L., Yue G., Yu J. (2022). Can the perceived risk of particulate matter change people's desires and behavior intentions?. Front. Public Health.

[20] Sass V., Kravitz-Wirtz N., Karceski S.M., Hajat A., Crowder K., Takeuchi D. (2017). The effects of air pollution on individual psychological distress. Health Place.

[21] Park I.R., Kim S. (2020). Response to risky society and searching for new governance: the role of risk communication factors in determining responding action for particulate matter. Korean Journal of Policy Analysis and Evaluation.

[22] Kim T. (1998). Valuing the health effects of air pollution. Korea journal of resource economics.

[23] Eom Y.S., Kim J.O., Ahn S.E. (2019). Measuring willingness to pay for PM 10 risk reductions: evidence from averting expenditures for anti-PM 10 masks and air purifiers. Environmental and resource economics review.

[24] Beck K.H. (1979). The effects of positive and negative arousal upon attitudes, belief acceptance, behavioral intention, and behavior. J. Soc. Psychol..

[25] Kim S., Lim C.H., Jeong J., Wang J.S., Park C.H. (2014). Analyzing the risk judgement about Fukushima nuclear accident and nuclear power by integrating the risk-perception paradigm with risk communication model. Korean J. Public Administration.

[26] Ajzen I., Fishbein M. (1973). Attitudinal and normative variables as predictors of specific behavior. J. Pers. Soc. Psychol..

[27] Fischhoff B., Watson S.R., Hope C. (1984). Defining risk. Pol. Sci..

[28] Slovic P., Fischhoff B., Lichtestein S., Warner F., Slater H. (1981). The Assessment and Perception of Risk.

[29] Kim S. (2021). Analysis of impact factors of trust in government under the crisis of covid-19: focusing on the change in determinant structure of trust in government by the difference from trusted objects and aggregation. J. Korean Assoc. Policy Stud..

[30] Ajzen I., Fishbein M. (1969). The prediction of behavioral intentions in a choice situation. J. Exp. Soc. Psychol..

[31] Shi H., Wang S., Zhao D. (2017). Exploring urban resident's vehicular PM2. 5 reduction behavior intention: an application of the extended theory of planned behavior. J. Clean. Prod..

[32] Fishbein M., Ajzen I. (1974). Attitudes towards objects as predictors of single and multiple behavioral criteria. Psychol. Rev..

[33] Morton C., Mattioli G., Anable J. (2021). Public acceptability towards Low Emission Zones: the role of attitudes, norms, emotions, and trust. Transport. Res. Pol. Pract..

[34] Shin Y., Kim S., Kim S. (2022). Searching for new human behavior model in the climate change age: analyzing the impact of risk perception and government factors on intention–action consistency in particulate matter mitigation. Int. J. Environ. Res. Publ. Health.

[35] Kim Y., Moon M.A. (2020). Study of the effects of government disaster management performance and trust in government on public engagement in social disaster preparedness. Korean J. Local Gov. Stud..

[36] Cho W., Im T., Porumbescu G.A., Lee H., Park J. (2013). A cross-country study of the relationship between Weberian bureaucracy and government performance. International Review of Public Administration.

[37] Cowell F.A., Gordon J.P. (1988). Unwillingness to pay: tax evasion and public good provision. J. Publ. Econ..

[38] Scholz J.T., Lubell M. (1998). Trust and taxpaying: testing the heuristic approach to collective action. Am. J. Polit. Sci..

[39] Blekesaune M., Quadagno J. (2003). Public attitudes toward welfare state policies: a comparative analysis of 24 nations. Eur. Socio Rev..

[40] Mettler S., Soss J. (2004). The consequences of public policy for democratic citizenship: bridging policy studies and mass politics. Perspect. Polit..

[41] Cherry T.L., Garcia J.H., Kallbekken S., Torvanger A. (2014). The development and deployment of low-carbon energy technologies: the role of economic interests and cultural worldviews on public support. Energy Pol..

[42] Wlezien C., Soroka S.N. (2016).

[43] Stadelmann-Steffen I., Eder C. (2021). Public opinion in policy contexts. A comparative analysis of domestic energy policies and individual policy preferences in Europe. Int. Polit. Sci. Rev..

[44] Park C.H., Kim S.Y. (2015). The role of knowledge in acceptance of nuclear power: a focus on objective and subjective knowledge. Korean Journal of Public Administration.

[45] Stoutenborough J.W., Sturgess S.G., Vedlitz A. (2013). Knowledge, risk, and policy support: public perceptions of nuclear power. Energy Pol..

[46] Ostrom E. (2019). Theories of the Policy Process.

[47] Churchland P.S., Sejnowski T.J. (1992).

[48] Kim S. (2015). Policy preferences, policy awareness, and the impact of policy voting: focusing on the 18th presidential election. Congressional Research.

